# Maternal Exposure to Ozone Modulates the Endophyte-Conferred Resistance to Aphids in *Lolium multiflorum* Plants

**DOI:** 10.3390/insects11090548

**Published:** 2020-08-19

**Authors:** Ludmila M. Bubica Bustos, Andrea C. Ueno, Tara D. Di Leo, Carlos D. Crocco, M. Alejandra Martínez-Ghersa, Marco A. Molina-Montenegro, Pedro E. Gundel

**Affiliations:** 1 IFEVA, CONICET, Cátedra de Ecología, Facultad de Agronomía, Universidad de Buenos Aires, Ciudad de Buenos Aires, Av. San Martín 4453, Argentina; aueno@agro.uba.ar (A.C.U.); dileo@agro.uba.ar (T.D.D.L.); martinez@agro.uba.ar (M.A.M.-G.); gundel@agro.uba.ar (P.E.G.); 2 IFEVA, CONICET, Cátedra de Fisiología Vegetal, Facultad de Agronomía, Universidad de Buenos Aires, Ciudad de Buenos Aires, Av. San Martín 4453, Argentina; ccrocco@agro.uba.ar; 3 Instituto de Ciencias Biológicas, Universidad de Talca, Av. Lircay S/N, Talca 3460000, Chile; marco.molina@utalca.cl; 4 Centro de Estudios Avanzados en Zonas Áridas, Universidad Católica del Norte, Coquimbo, Ossandón 877, Chile; 5 Centro de Investigación en Estudios Avanzados del Maule, Universidad Católica del Maule, Talca, Av. San Miguel 3605, Chile

**Keywords:** transgenerational effects, maternal effects, fungal endophyte, symbiosis, *Rhopalosiphum padi*, *Lolium multiflorum*

## Abstract

**Simple Summary:**

Global change is driving the incidence of novel stress factors such as the tropospheric pollutant ozone. Plants can overcome the environmental challenges by adjusting their phenotypes that eventually, can be transmitted to the progeny. Plants also establish symbiotic interactions with beneficial fungal endophytes, some of which can be transmitted to the progeny through the seeds. We worked with the grass *Lolium multiflorum* and its common endophyte, *Epichloë occultans* that benefits the host by coffering resistance to herbivores. Specifically, we studied the effect of exposing endophyte-symbiotic and non-symbiotic plants to ozone on the level of resistance to herbivorous aphids in the progeny. The ozone history impaired the endophyte-conferred resistance to aphids in progeny. This was evident at individual weight level of aphids but not so at population level. Defensive compounds were higher in endophyte-symbiotic seeds but depressed by mother plants exposure to ozone. Despite the negative effect of maternal ozone on the resistance level of plants, symbiotic plants showed a superior biomass compared to endophyte-free plants. Our work illustrates how the environment explored by parent plants can persist transgenerationally and, depending on the symbiotic state, will affect the fate of the progeny.

**Abstract:**

Plants are challenged by biotic and abiotic stress factors and the incidence of one can increase or decrease resistance to another. These relations can also occur transgenerationally. For instance, progeny plants whose mothers experienced herbivory can be more resistant to herbivores. Certain fungal endophytes that are vertically transmitted endow plants with alkaloids and resistance to herbivores. However, endophyte-symbiotic plants exposed to the oxidative agent ozone became susceptible to aphids. Here, we explored whether this effect persists transgenerationally. We exposed *Lolium multiflorum* plants with and without fungal endophyte *Epichloë occultans* to ozone (120 or 0 ppb), and then, challenged the progeny with aphids (*Rhopalosiphum padi*). The endophyte was the main factor determining the resistance to aphids, but its importance diminished in plants with ozone history. This negative ozone effect on the endophyte-mediated resistance was apparent on aphid individual weights. Phenolic compounds in seeds were increased by the symbiosis and diminished by the ozone. The endophyte effect on phenolics vanished in progeny plants while the negative ozone effect persisted. Independently of ozone, the symbiosis increased the plant biomass (≈24%). Although ozone can diminish the importance of endophyte symbiosis for plant resistance to herbivores, it would be compensated by host growth stimulation.

## 1. Introduction

Global climate change is regularly associated with increasing land degradation and contamination of air and water sources; hence, it negatively affects biodiversity and ecosystem functioning [1]. How plants and their interacting species, either beneficial (e.g., fungal symbionts) or detrimental (e.g., herbivores and pathogens), respond to these challenges is fundamental to predict the impact of global change [2,3]. Together with the capacity of plants to adjust their phenotype, it is key to understand how environmental factors of global change can disrupt synchronicity in trophic relationships [4,5,6,7]. While most studies addressing plant-herbivore interaction under global change scenarios have focused on CO_2_ and temperatures, a few works have considered other abiotic stressors such as ozone [8,9]. Ground-level ozone is a pollutant rising globally that, mainly associated to human activities, causes oxidative damage in animals, plants, and microorganisms [10]. While research has commonly focused on the impact of one stress factor on the interaction of plants with other organisms during the host ontogeny, recent attention is moving to understand the legacy effects of global change factors on the progeny ecological performance [11,12,13]. 

Transgenerational effects (TGE) refer to phenotypic variation expressed in the progeny that is explained by the environmental conditions experienced by maternal plants [14,15,16,17]. A recent review paper clearly show that TGE are stronger in annual plants and, generally enhance fitness of the progeny in response to stressful environmental conditions [13]. Maternal plants contribute with seed coat, nutrition, plastids, and metabolites, while epigenetic regulation of DNA seems to play a major role in determining long-term effects on the future plant phenotype [11,14,18,19,20,21]. In addition, certain plants can transmit microbial symbionts to the progeny, which can eventually play a role in offspring fitness [22,23,24]. However, the potential contribution of vertically transmitted microbial symbionts to TGE of plant in response to environmental stress factors is basically unexplored [25,26,27]. 

A very interesting biological system to study the role of symbiotic microorganisms on TGE in plants is the one established between certain cool-season grasses and vertically transmitted fungal endophytes [26]. The fungal endophytes of the genus *Epichloë* (Ascomycota: Clavicipitaceae) are endosymbionts of grasses (family Poaceae) that establish an asymptomatic and beneficial interaction [28,29]. Asexual fungal species grow in the apoplast of the plant green tissues and transmit vertically by growing hyphae into the developing seeds [24,28,29]. The endophytic mycelium as well as fungal secondary metabolites such as the alkaloids are found in the seed (in the embryo, around cells in the aleurone layer and close to the scutellum) [27,30,31,32]. Of the various benefits delivered by the fungal endophytes to host grasses, the protection against herbivores, which is explained by the existence of the fungal alkaloids, is by-far the most prominent one [28,33,34]. In addition to the alkaloid-mediated herbivore resistance, endophytes can be source of other metabolites with functional roles such as phenolic compounds [35,36,37]), antioxidants [38,39,40] and phytohormones [41]. These are suggested to explain the usually documented higher performance of endophyte-symbiotic plants, relative to endophyte-free plants, under conditions of stress caused by biotic (competing plants, herbivores, pathogens) and abiotic factors (drought, heavy metal, herbicides) [38,39,40,42,43,44,45]. Nonetheless, the endophyte effects on host plant performance have been shown to depend on the ecological context and thus, symbiosis outcome can yield positive, neutral, or even negative [46,47,48]. 

Ozone is an air pollutant and a potent oxidant for living organisms [10]. The concentration of ground-level ozone is rising linked to human activities, causing damage to natural and managed ecosystems [8,10,49,50]. In plants, ozone enters through stomata and reacts with cell structures, triggering the accumulation of Reactive Oxygen Species (ROS) [51]. This oxidative stress is, to some extent, counteracted by the plant antioxidant system [52,53,54]. Due to its variable dynamics in space and time, plants can be exposed to ozone for short (episodic) or long (chronic) periods of time [10]. It has been suggested that it can increase plant resistance to herbivores and pathogens by means of activating specific hormone signaling pathways (e.g., Salicylic Acid defense pathway; see [55]) and/or by promoting the synthesis and accumulation of secondary metabolites such as phenolic compounds, terpenoids, and antioxidants [55,56,57,58,59,60]. For example, resistance to the aphid *Myzus persicae* S. on *Eruca sativa* Mill. was increased by exposing plants to a few episodes of ozone [61]. Recently, it was shown that 5 days—but not 1 day—of exposure to ozone increased resistance to the herbivorous larvae of *Pieris brassicae* L. in plants of *Sinapis arvensis* L. (wild mustard) [62]. On the contrary, we found that the endophyte-conferred resistance to the herbivorous aphid *Rhopalosiphum padi* L. in *Lolium multiflorum* Lam. was disrupted by exposing plants to very short episodes (2 days, 2 h per day) of high concentration of ozone, even though ozone treatment increased the resistance level in endophyte-free plants [9]. As a widely diverse group of multifunctional molecules [63], phenolic compounds could link the plant exposure to ozone with the resistance to herbivores as they are well-known to play defense roles in seeds as well as in vegetative organs; even though their role may be different depending on the plant phenological stages [53,57,64,65,66]. However, no attempts have been made trying to establish this link. Therefore, besides the assumption that ozone effects on the plant-herbivore interaction may depend on the underlying resistance mechanism (that is, whether it is of the plant or modulated by symbiotic microorganisms), it is certainly unknown whether these effects can be transmitted to the next generation. 

Here, we tested the ozone transgenerational effect on plant resistance to herbivory and how this can be mediated by fungal endophytes of vertical transmission. In particular, we might expect different effects of the maternal ozone legacy on endophyte-symbiotic and non-symbiotic plants. First, ozone can induce resistance to aphids on exposed plants, a state that eventually will be expressed in the progeny. Second, as the fungal endophyte can be the target of ozone-induced stress and the defensive mutualism impaired, as we observed early [9], the endophyte-conferred resistance to aphids is spoiled in progeny plants. In our previous study [9], we observed that ozone impairment of the defensive mutualism was not associated to a low concentration of alkaloids. Therefore, we focused here on phenolic compounds because (i) they can have multiple effects in protecting plants against oxidative stress and biological threats [64,67,68], (ii) they are induced in plants exposed to ozone [53,57], and (iii) they are enhanced in plants symbiotic with *Epichloë* fungal endophytes [35,36,37]. We attempted to establish a link among ozone-induced TGE, the presence of vertically transmitted fungal endophytes, and plant resistance to herbivores by following the variation in total phenolic compounds in seeds and progeny plants. 

## 2. Materials and Methods 

### 2.1. Biological Material 

We worked with the persistent symbiosis established between *Lolium multiflorum* Lam. (Poaceae), commonly known as Italian ryegrass, and its fungal endophyte *Epichloë occultans* (C.D. Moon, B. Scott & M.J. Chr.) Schardl. [69,70]. Italian ryegrass is a high-quality forage grass native to Mediterranean that became naturalized in the Pampa grasslands of Argentina. It is a self-incompatible and wind-pollinated annual species with C_3_ metabolism. Pampean populations are known to exhibit high frequency of endophyte symbiotic individuals infected with *E. occultans* [34,71]. In this work, we used an endophyte-infected population (E+) and the endophyte-free counterpart (E−). Plants from these two biotypes differ in their infection status but belong to the same population [72].

We collected *R. padi* L. (Hemiptera: Aphididae) from the wild in October 2016. Commonly known as bird cherry-oat aphid, it is a phytophagous insect able to infest a wide range of cereals and grasses [73]. First, the collected aphids were maintained for five days in Petri dishes with pieces of fresh leaves of oat (*Avena sativa* L.); during this period, parasitized individuals were removed. After that, 10 aphids (5 nymphs and 5 apterous adults) were placed and reared on young oat plants [74]. The oat plants and aphids were in a growth chamber with controlled environmental conditions (Temperature: 23 °C (±1), photoperiod: L16:D8 h, y radiation: 150 μmol m^−2^ s^−1^). Five to six oat plants were grown in 1 L pots filled with a commercial potting-mix substrate and no water deficit. Every time the oat plants showed symptoms of deterioration, they were renewed. The number of individuals and the proportion of different instars (nymphs, apterous, and winged adults; *sensu* [75]) was assessed every day. In the experiments presented here, we only worked with apterous adult aphids. 

### 2.2. Experimental Design

The experiment was carried out in an experimental field and the ozone-chamber facilities at the School of Agronomy, University of Buenos Aires (Argentina). The whole experiment involved first, the generation of maternal endophyte-symbiotic and non-symbiotic plants with different history of exposure to ozone and second, the evaluation of the progeny performance in relation to herbivory by aphids. 

#### 2.2.1. History of Maternal Plants Exposure to Ozone

**Figure 1:**
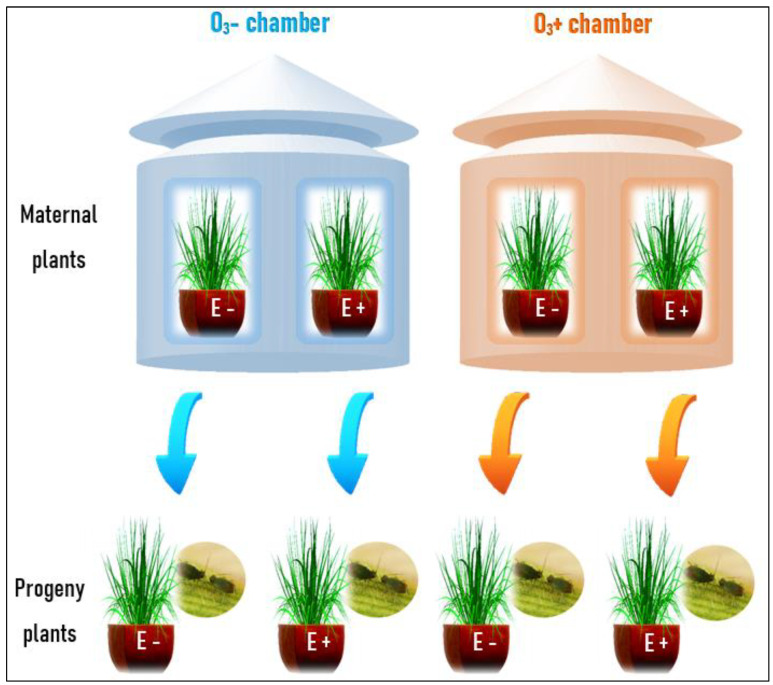
Scheme of the experimental design. The upper part of the figure represents the open-top chambers in which ozone was manipulated [without ozone ≈ 0 ppb (O_3_^−^) and with ozone ≈ 120 ppb (O_3_^+^)] and *Lolium multiflorum* maternal plant symbiotic (E+) and non-symbiotic (E−) with the fungal endophyte *Epichloë occultans*. The lower part shows the progeny plants and herbivory treatment by the aphid *Rhopalosiphum padi* for 21 days. The arrows indicate the maternal ozone history (light blue is O_3_^−^ and orange is O_3_^+^).

In 2015, we grew *L. multiflorum* plants with (E+) and without (E−) *E. occultans* individually in three L pots filled with a commercial potting-mix. The plants were kept outdoors and watered on demand. At flowering, they were exposed to two contrasting concentrations of ozone [high level (120 ppb) and low level (0 ppb)] in open-top chambers (see [9]) (Figure 1, Figures S1 and S2). Each chamber is a vertical cylinder (2 m diameter) with a metal skeleton is embraced by a transparent PVC plastic wall (Supplementary Materials). Ozone is produced by a spark discharge-type generator (OZ5000, Dobzono, AR), that is differentially delivered between chambers through a pipe system. Each pipe reaching each chamber is regulated by a valve and mixed with a constant flow of activated carbon-filtered air. Ozone concentration within each chamber is monitored using a model 450 ozone monitor API-Teledyne Instrument (Teledyne Advanced Pollution Instrumentation, Inc., San Diego, CA, USA). Six plants of each symbiotic biotype (E+ and E−) were randomly assigned to any of the 8 open-top chambers (4 with high and 4 with low-ozone concentration) (96 plants total). The treatment lasted three weeks and plants were exposed to ozone four hours per day (from 11 a.m. to 3 p.m.), to simulate the daily natural dynamics of ozone in nature, which is basically associated to the peak of temperature and radiation [10]. After the treatment, the plants were returned to the experimental field, randomly placed, and were maintained until reaching the maturity state. The seeds produced by all the plants from a given symbiotic status within a chamber, were pooled, and stored in dry and cold (~5 °C) condition. Before pooling the seeds, each plant was evaluated to confirm its symbiotic status. Ten seeds per plant was examined under light microscope (100×) previously stained with Bengal rose and squashed on a coverslip [32,76]. Therefore, we only pooled plants from each chamber, with 100% endophyte positive or 100% endophyte negative as endophyte-symbiotic (E+) or endophyte-free (E−), respectively. These seeds were used for the next part of the experiment, progeny plants. 

#### 2.2.2. Transgenerational Effect of Ozone on Progeny Plant Resistance to Aphids 

In 2016, the progeny plants were characterized for their resistance level to the aphid *R. padi*. Twenty symbiotic and twenty non-symbiotic seeds from each ozone condition (O_3_^−^ and O_3_^+^) were directly sown in 670 mL pots filled with the same substrate as before, and placed in a grid outdoors (with positions randomly assigned), *n* = 20. When the plants had 3–4 tillers, all of them were challenged by 10 apterous adult aphids (Figure 1). To keep the aphids on the plants and to avoid others from attacking the plants, each one was surrounded with a plastic net cylinder (2 cm^2^ mesh) and the outer part covered by white fabric (0.05 mm mesh) (Figure S3). The aphids were kept on the plants for 21 days.

After that period, we characterized the performance of aphids to evaluate the level of resistance of plants due to maternal ozone history and endophytic symbiosis. First, all individuals that were in a plant were collected to estimate population size and structure. They were placed in a Petri dish, and homogeneously distributed. The Petri dish was divided in six parts of equal-size, and the aphids in 1/6 were carefully counted and classified per instar. For better visualization, this procedure was carried out using a Binocular stereoscopic microscope. Population structure was assessed by estimating the proportion of individuals in each instar: nymphs, apterous, and winged adults [75]. The number and proportion in each instar determined for 1/6 was extrapolated to the whole dish (i.e., plant). Second, to estimate individual mean weight, we randomly took 40 aphids per instar that were weighted in a laboratory precision balance (Denver Instruments, model APX-200, ±0.0001 g) and then, divided by 40. For a better presentation, the aphid weights are expressed in µg.

Finally, the aboveground biomass of each plants was individually harvested and placed inside a paper bag. Then, the bags were stored in an oven to dry (60–70 °C). After being there for about 72 h, the material was weighed on a laboratory balance (±0.001 g) to obtain dry weight.

### 2.3. Determination of Phenolic Compounds 

Concentration of total phenolic compounds was determined in both, seeds produced by mother plants, and in leaf tissue sampled from progeny plants. In the first case, we used five seeds per plant (12 plants/treatment). The seeds were ground into liquid nitrogen until we obtained a white powder. Each sample was then transferred to a 1.5 mL plastic tube. Methanol 800 µL: HCl 1% was added and the tube was put into ice. All tubes were stored for 48 h/ at −20 °C in the dark. Samples were placed during 30 s in the vortexer and one minute into the centrifuge. Total phenolic compounds were quantified with spectrophotometry techniques, measuring absorbance from the samples at 305 nm and 320 nm. For that purpose, we took 600–700 uL; from the supernatant liquid and methanol: HCl 1% solution was used as blank sample, adapted from [77]. In the second case, total phenolic compounds were determined in a circular piece of healthy leaf blade taken with a puncher (6 mm diameter) of progeny plants. The disc was taken 4 mm from the ligule (the intersection between the sheath and blade). Similarly, each leaf disc was put into a plastic tube (1.5 mL) with 800 uL methanol: HCl 1%, stored in the −20 °C. After 48 h, total phenolic compounds were indirectly determined by spectrophotometry, following the same procedure described above for the seed samples. 

### 2.4. Data Analysis

All statistical analysis was performed using the software R [78] and when corresponded for any model, we used Tukey’s tests (*p* < 0.05) to highlight differences between treatments. Phenolic compounds concentration was analyzed using linear mixed models with normal distribution (package nlme; [79]). The models included the plant symbiotic status (E+, E−) and maternal ozone treatment (O_3_^+^, O_3_^−^) as fixed factors and the ozone chamber as random factor. To accommodate deviations in variance homogeneity, VarIdent variance structure was used on plant symbiotic status [80]. Akaike Information Criterion (AIC) was used to compare the models for each response variable and to choose the best-adjusted models [80]. 

The aphid population size (number of aphids per individual plant) was analysed with generalized linear mixed models using Poisson distribution (package lme4; [81]) where the plant symbiotic status (E+, E−) and maternal ozone treatment (O_3_^+^, O_3_^−^) were using as fixed factors and the ozone chamber as random factor. The structure of aphid populations was analysed with a generalized linear model using binomial distribution (nymph and adults aphid categories) and logit link function with the lme4 package [81]. The model included the symbiotic status (E+, E− plants) as categorical/fixed factor. Model selection was based on Chi-test nested models and dispersion parameter (phi) was calculated to evaluate the fit (or adequacy) of the model. Data overdispersion was not detected. 

The plant biomass was analysed using general linear model with normal distribution of the error, with the package nlme [79]. The model included the plant symbiotic status (E+, E−) and ozone treatment (O_3_^+^, O_3_^−^) as fixed effects and the ozone chamber as random factor. To accommodate deviations in variance homogeneity, VarIdent variance structure was used on plant symbiotic status [80]. Akaike Information Criterion (AIC) was used to compare the models for each response variable and to choose the best-adjusted models [80]. ANOVA results of the selected models are only shown. All values are means ± SE of the mean.

## 3. Results

### 3.1. Transgenerational Effect of Ozone on the Resistance Level of Progeny Plants 

**Figure 2:**
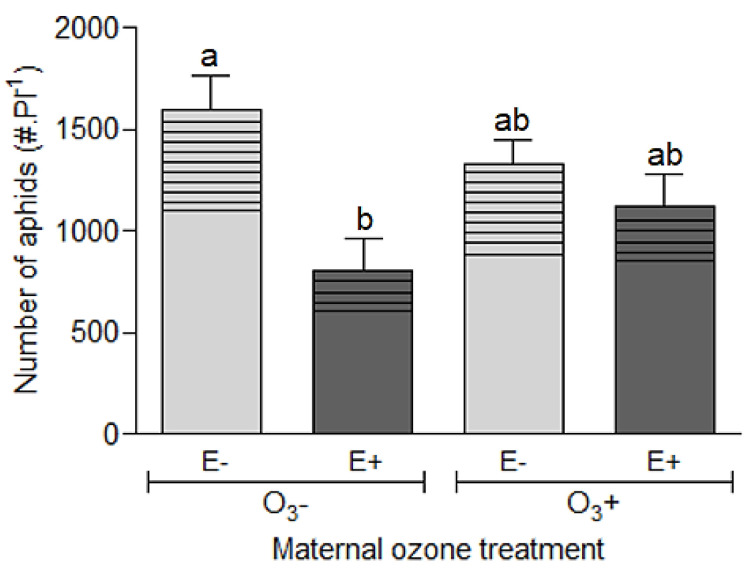
Population size (number of aphids per plant) and population structure [proportion of individuals per instar: “nymphs” (no pattern design) and “apterous + winged adults “ (with pattern design)] of the aphid *Rhopalosiphum padi* after growing for 21 days on *Lolium multiflorum* progeny plants symbiotic (E+) and non-symbiotic (E−) with fungal endophyte *Epichloë occultans*, whose mother plants were exposed to tropospheric ozone [with ozone ≈120 ppb (O_3_^+^)] or not [without ozone ≈0 ppb (O_3_^−^)]. Different letters indicate significant differences based on Tukey’s test at <0.05. For total number of aphids, values are means ± S.E. (*n* = 20).

The aphid population size after 21 days feeding on progeny plants was mainly affected by the presence of the fungal endophyte (χ^2^ = 10.372, *p* = 0.001), independently of the mother plant exposure to ozone (χ^2^ = 3.059, *p* = 0.080). However, the endophyte effect on aphid performance was much more pronounced in progeny plants without history of ozone exposure than in plants with history of ozone exposure (Figure 2 and Table S1). The average number of aphids per plant without maternal ozone treatment was 50% lower in symbiotic plants (E+) than non-symbiotic plants, while this difference was reduced in a ≈15% in plants with history of ozone (Figure 2). The history of maternal plant exposure to ozone did not affect the aphid population size (χ^2^ = 1.844, *p* = 0.174). 

The aphid population structure, that is the relative number of nymphs and adults (apterous + winged), was affected by the symbiotic status of the plants with fungal endophytes (χ^2^ = 9.600, *p* = 0.001), independently on the exposure of maternal plants to ozone (χ^2^ = 0.001, *p* = 0.957) (Figure 2 and Table S2). The number of nymphs was on average 27% lower in E+ plants than in E− plants. On the other hand, the number of adult aphids was 49% higher in E− plants with respect to that in E+ plants.

The effect of the treatments on aphid individual weights varied depending on the instar. For nymphs and apterous adults, the individual weight was affected by an interaction between the symbiotic status of plants and the maternal history of ozone exposure (Nymphs: F_1, 67_ = 6.630, *p* = 0.012; Apterous adults: F_1, 65_ = 4.071, *p* = 0.048) (Table 1 and Table S3). The negative impact of the endophyte presence on individual weight of the nymphs (≈39% lower) in control plants (no maternal ozone exposure) was completely inverted by the maternal history of ozone exposure (≈21% higher). In a similar way, the strong negative effect of endophyte on individual weight of apterous adults (31% lower) vanished on plants with maternal history of ozone exposure (Table 1 and Table S3). By contrast, the individual weight of winged aphids was not affected by either the symbiotic status of plants (F_1, 37_ = 0.010, *p* = 0.919) nor the maternal history of ozone (F_1, 37_ = 0.134, *p* = 0.726). 

### 3.2. Transgenerational Effect of Ozone on the Defense Level of Progeny Plants 

**Figure 3:**
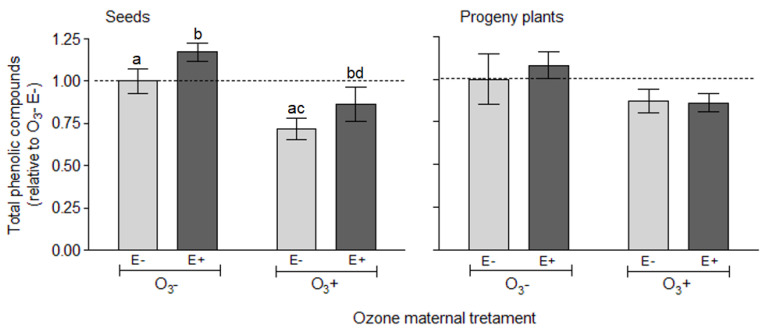
Variation in total phenolic compounds in seeds and progeny plants of *Lolium multiflorum* plants symbiotic (E+) and non-symbiotic (E−) with fungal endophyte *Epichloë occultans*, whose mother plants were exposed to tropospheric ozone [with ozone ≈ 120 ppb (O_3_^+^)] or not [without ozone ≈ 0 ppb (O_3_^−^)]. Values are expressed relative to control condition [no endophyte (E−) and no ozone (O_3_^−^)] for each category independently. Different letters indicate significant differences (Tukey’s test, *p* < 0.05); lack of letters in a response variable means there were no significant effects. Values are means ± S.E. (*n*_seeds_ = 12, and *n*_plants_ = 23).

The concentration of total phenolic compounds in the seed was independently affected by the plant symbiotic status (F_1, 38_ = 5.259, *p* = 0.027) and mother plant exposure to ozone (F_1, 6_ = 8.210, *p* = 0.028). Seeds from symbiotic plants presented on average a higher concentration of phenolic compounds (≈18%) than seeds from non-symbiotic plants (Figure 3, Seeds and Table S4). The mother plant exposure to ozone caused a reduction in total phenolic compounds in the seed by about 27% (Figure 3, Seeds).

Despite a clear tendency for progeny plants coming from mothers exposed to ozone to present lower concentration of phenolic compounds (≈17% lower), the effects of the manipulated factors were not statistically significant (Figure 3, progeny plants and Table S4). In particular, the concentration of total phenolic compounds in progeny plants was neither affected by the history of exposure to ozone (F_1, 6_ = 0.211, *p* = 0.662) or by the symbiotic status with endophytes (F_1, 70_ = 1.512, *p* = 0.222). 

**Figure 4:**
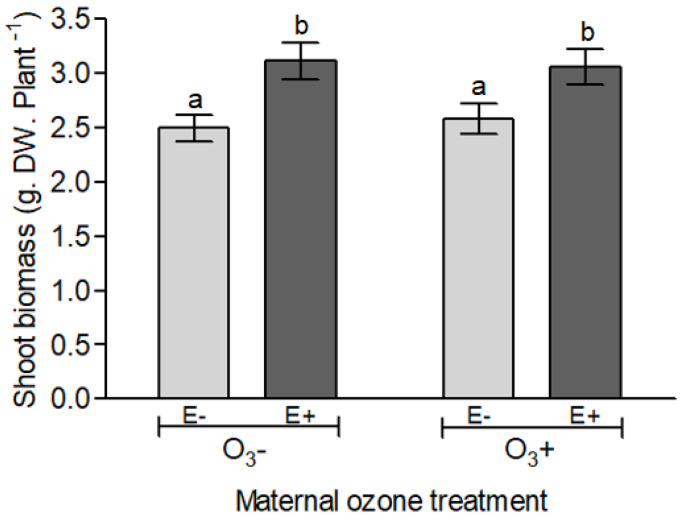
Shoot biomass (g dry weight) of *Lolium multiflorum* progeny plants symbiotic (E+) and non-symbiotic (E−) with fungal endophyte *Epichloë occultans*, whose mother plants were exposed to tropospheric ozone [with ozone ≈120 ppb (O_3_^+^)] or not [without ozone ≈0 ppb (O_3_^−^)] at the experiment (21 days after the herbivory treatment with *Rhopalosiphum padi*). Different letters indicate significant differences among means (Tukey’s test, *p* < 0.05). Values are means ± S.E. (*n* = 20).

The aboveground biomass of progeny plants was mainly controlled by the symbiotic status of plant (F_1, 68_ = 11.761, *p* = 0.001) and not dependent on the mother plant exposure to ozone (F_1, 68_ = 0.630, *p* = 0.422). On average, endophyte-symbiotic plants presented 22% more biomass than non-symbiotic plants (Figure 4 and Table S5). 

## 4. Discussion

We showed that ozone applied on *L. multiflorum* maternal plants drove changes in the interaction of progeny plants with the herbivorous aphids. Interestingly, the change depended on the plant symbiotic status with leaf fungal endophytes. The transgenerational ozone impaired the plant resistance to sap-sucking insects conferred by fungal endophytes. Although endophyte-free progeny plants from ozone-exposed mother plants tended to display higher resistance to aphids than those from non-exposed mother plants, this was not supported by the concentration of total phenolic compounds. Although it seems not to easily explain the pattern of plant herbivore resistance, the transgenerational ozone diminished the concentration of total phenolic compounds in progeny seeds and plants. Independently of the ozone history, the symbiosis with fungal endophytes clearly benefited host plants by boosting vegetative growth. 

We evaluated the level of plant resistance to herbivory by looking at the effects of the manipulated factors (i.e., symbiosis with *Epichloë* fungal endophytes and the legacy of maternal plant exposure to ozone) on aphids’ performance. The total number of aphids per *L. multiflorum* plant was significantly reduced by the presence of the fungal endophyte *E. occultans,* indicating a higher resistance level; which is in agreement with previous studies conducted with the same symbiotic system and herbivore species [9,72,82]. This difference in resistance level was much more evident in plants without history of ozone that in plants with history of ozone (actually, there was a marginal interaction effect; *p*-value = 0.08). In addition to population size, changes in population structure can give information related to food quality and herbivore potential growth. For example, high proportion of nymphs may be indicative of good conditions for growth and reproduction. Alternatively, a low proportion of nymphs and a high of adults may indicate low food availability (low carrying capacity) or the presence of toxic elements that can be perceived as cue for abandon the plant (associated with high proportion of winged adults) [63,82,83,84,85]. If the population has reached the carrying capacity or the food quality is poor, winged adults will leave the plant in search of a new one [83]. Here, we observed strong endophyte effect on the population structure, but it was irrespective of the maternal plant ozone treatment. Typically, the presence of *E. occultans* in *L. multiflorum* plants produced populations of aphids with high adult/nymph ratio [9,82]. This indicates that aphid populations are reaching the carrying capacity (see [83,84]) in endophyte-symbiotic plants with lower number of individuals. In summary, these two population-level response variables—size and structure—that were strongly affected by the endophyte symbiont, were not significantly affected by the transgenerational ozone effect.

The effects of transgenerational ozone on the differential level of herbivory resistance between E+ and E− *L. multiflorum* progeny plants were detected at the individual level. In line with previous results [9,82], there was a clear reduction in the individual weight of aphids reared on plants with fungal endophytes, which was evident on nymphs and apterous adults, but not on winged aphids. However, there was no endophyte effect on individual weight of aphids when they were reared on progeny plants with history of ozone. Therefore, there was an endophyte-mediated mechanism conferring plant resistance to aphids which was impaired by the transgenerational effect of ozone. Interesting to note is that when these effects at individual level are scaled up to population (by multiplying individual mean weight by the number of aphids in each instar), there is an endophyte effect on the resistance level to herbivores that is significant or negligible in plants without (control) or with ozone legacy, respectively. *Epichloë* fungal endophytes are known to endow plants with a herbivory resistance mechanism by means of producing bioactive alkaloids [33,86,87]. However, the impairment of the endophyte-conferred resistance mechanism against aphids due to a direct exposure of plants to ozone was not mediated by a reduction in the loline alkaloids [9]. Besides the alkaloids, it is suggested that fungal endophytes would increase the resistance level of host plants by synthetizing secondary metabolites and by boosting the plant’s own immune system [35,36,37,45,82,88]. Here, although total phenolic compounds were higher in endophyte-symbiotic seeds, suggesting a higher defense level against stress and herbivores [64], this effect vanished in the progeny plants. Therefore, it did not explain the variation in the level of resistance to the aphid *R. padi* in endophyte-symbiotic *L. multiflorum* plants. 

The ozone can modify the profile of produced defense compounds by both, the plants and the fungal endophytes, inducing the synthesis of secondary metabolites such as phenolic compounds [53,56,57,89]. Here, we focused on total phenolic compounds as a potential link between ozone and plant resistance to herbivory. Phenolic compounds can be induced in plants exposed to ozone [53,57] and to play a defensive role against biotic and abiotic threats [64,67,68,90,91]. Contrary to expectations, we found that ozone caused a significant depression in the content of phenolic compounds in seeds; in spite of a clear tendency for a similar effect in the progeny plants, either endophyte-symbiotic or non-symbiotic ones, the latter was not significant. Together with the endophyte effects (see [37,45]), the profile and abundance of phenolic compounds can be different among plant organs or phenological stages [64,65,66]. Although total phenolic compounds were not affected by the endophyte presence within a given treatment of ozone, it is possible that specific groups of phenols had responded differently [89]. For example, different treatments of ozone exposure caused differential response in tannins and flavonoids in plants of *Tibouchina pulchra*, a variation that was not perceived in total phenolic compounds [92]. The induction of defense compounds by ozone [55,56,57] could be passed on to the progeny through epigenetic mechanisms [11,12]; but our results do not support this. What is more intriguing is that the ozone applied on mother plants had no consequences on the accumulated biomass of progeny plants. In terms of plant biomass, the symbiosis with fungal endophytes promoted the accumulation of aboveground tissues significantly and irrespective of the ozone history. The fungal endosymbiont benefits on the plant growth have been well documented in other symbiotic systems [28] and in *L. multiflorum—E. occultans* (e.g., [9,72,82,93]). Therefore, despite the endophyte-conferred resistance to herbivores have been impaired by the ozone (during the ontogeny and transgenerationally), there was a marked benefit of the endophyte symbiosis on host plants as they accumulated more vegetative biomass that the endophyte-free counterparts. 

Transgenerational memory in plants refers to any environmental factor-induced effect that is stored and transmitted to the progeny [13,19,86,94,95]. These TGEs may influence not only the performance of progeny plants but also the ecological interactions with microorganisms and animals [13,15,16,26,96]. In relation with our study system, we had previously demonstrated that the *E. occultans* endophyte-conferred resistance to the aphid *R. padi* in *L. multiflorum* plants was negatively affected by just a short event of high ozone [9]. The present study suggests that this negative effect could persist in a transgenerational way. Unlike our work, most studies on TGE in plants evaluate the impact of a stress factor on parental plants and the performance of the progeny facing the same factor [13]. Therefore, in our work, the transgenerational response of plants to ozone might not have been so straightforward because (i) we studied the consequences of an abiotic maternal stress factor on the resistance to herbivory in the offspring, and because (ii) the responses of mother plants to the abiotic stress factor and the consequences on the progeny resistance to herbivores were mediated by a symbiotic microorganism. Therefore, our work highlights that future research on plant transgenerational effects should explicitly control the symbiotic status of hosts to unveil the precise mechanisms underlying a given type of response. 

## 5. Conclusions

Here we showed that ozone effects on plant resistance to herbivory persisted on the progeny and depended on the endophyte symbiotic status. Interestingly, while ozone transgenerational effects tended to increase the plant resistance to aphids in endophyte-free plants, the opposite was observed on endophyte-symbiotic plants. The resistance to herbivorous aphids conferred by *Epichloë* fungal endophytes was not observed in ozone-exposed plants, a susceptibility that persisted in the progeny plants. We found no evidence for total phenolic compounds to play role in linking the ozone exposure with plant resistance to herbivores. However, the positive effect of the fungal symbiont on plant growth prevailed over ozone, and despite of the herbivores. Our results highlight the complexity behind the legacy effects of one novel stress factor on the trophic interactions between plants, fungal endophytes, and herbivores. 

Future research should attempt to unveil the transgenerational effects of ozone not only on the plants but also on the fungal endophytes. Aspects such as in-plant hyphae density and alkaloid concentration can give insight on the affected processes that could explain the negative consequences and its transgenerational persistence of ozone pollution. Studies using molecular approaches could shed light into the hormonal pathways involved in the regulation of the plant-endophyte interaction when facing stressful conditions. Besides elucidating endophyte-mediated alternative regulation circuits to the plant immune system and production of secondary metabolites with specific defense roles, it will help to understand the underlying basis of the context dependency of symbiotic outcomes.

## Figures and Tables

**Table 1:** Individual fresh weight per instar (nymph, apterous adults, and winged adults) of the aphid *Rhopalosiphum padi* after grown for 21 days on *Lolium multiflorum* plants symbiotic (E+) and non-symbiotic (E−) with fungal endophyte *Epichloë occultans*, whose mother plants were exposed to tropospheric ozone [with ozone ≈ 120 ppb (O_3_^+^) and without ozone ≈ 0 ppb (O_3_^−^)] for three weeks (4 h/day). Different letters indicate significant differences (*p* < 0.05); lack of letters in a response variable means that there were no significant effects. Values are means ± S.E. (*n* = 20).

Treatments		Aphid Individual Weight (µg)		
Maternal Ozone	Endophyte	Nymphs	Apterous	Winged
O_3_^−^	E−	48 ± 6 (a)	100 ± 10 (a)	69 ± 5
	E+	31 ± 3 (b)	69 ± 6 (b)	68 ± 6
O_3_^+^	E−	38 ± 4 (ab)	92 ± 10 (ab)	66 ± 5
	E+	46 ± 9 (ab)	93 ± 9 (ab)	47 ± 5

## Supplementary Materials

The following are available online at https://www.mdpi.com/2075-4450/11/9/548/s1: Figure S1: Inside view of an open-top tropospheric ozone chamber with the *Lolium multiflorum* mother plants during the ozone exposure treatment. Figure S2: View of two of the eight open-top chambers used for the controlled ozone exposure of *Lolium multiflorum* plants in the experiment. Figure S3: View of the experimental setting where *Lolium multiflorum* plants with different symbiotic status and maternal ozone history were challenged with the aphid Rhopalosiphum padi during the herbivory treatment. The plants were surrounded by a plastic net cylinder and covered by a white fabric. Table S1: Significance of the effects of the endophyte symbiosis and mother plant exposure to ozone on response variable “number of aphids” over *Lolium multiflorum* progeny plants. Statistically significant effects are highlighted in bold. Mean values and S.E. and statistical differences are shown in Figure 2. Table S2: Significance of effects of the endophyte symbiosis and mother plant exposure to ozone on response variable “proportion of individuals per instar” over *Lolium multiflorum* progeny plants. Statistically significant effects are highlighted in bold. Mean values and S.E. and statistical differences are shown in Figure 2. Table S3: Significance of effects of the endophyte symbiosis and mother plant exposure to ozone on response variable “aphid individual weight” over *Lolium multiflorum* progeny plants. Statistically significant effects are highlighted in bold. Mean values and S.E. and statistical differences are shown in Table 1. Table S4: Significance of effects of the endophyte symbiosis and mother plant exposure to ozone on response variable “total phenolic compounds” over *Lolium multiflorum* progeny plants. Statistically significant effects are highlighted in bold. Mean values and S.E. and statistical differences are shown in Figure 3. Table S5: Significance of effects of the endophyte symbiosis and mother plant exposure to ozone on response variable “shoot biomass” over *Lolium multiflorum* progeny plants. Statistically significant effects are highlighted in bold. Mean values and S.E. and statistical differences are shown in Figure 4.
