# Supplementary material for: Maternal Exposure to Ozone Modulates the Endophyte-Conferred Resistance to Aphids in Lolium multiflorum Plants
Source: Insects. 2020 Aug 19;11(9):548. doi: 10.3390/insects11090548 (PMC7564161; doi:10.3390/insects11090548)
Supplement: Supplementary file 1 [file insects-11-00548-s001.pdf]

Maternal exposure to ozone modulates the endophyte-conferred resistance to aphids in *Lolium multiflorum* plants

Ludmila M. Bubica Bustos[1]*, Andrea C. Ueno[1], Tara D. Di Leo[1], Carlos D. Crocco[2], M. Alejandra Martínez-Ghersa[1], Marco A. Molina-Montenegro[3, 4, 5] & Pedro E. Gundel[1, 3]

[1] IFEVA, CONICET, Cátedra de Ecología, Facultad de Agronomía, Universidad de Buenos Aires, Ciudad de Buenos Aires, Av. San Martín 4453, Argentina; aueno@agro.uba.ar (A.C.U.); dileo@agro.uba.ar (T.D.D.L.); martinez@agro.uba.ar (M.A.M.-G.); gundel@agro.uba.ar (P.E.G.)
[2] IFEVA, CONICET, Cátedra de Fisiología Vegetal, Facultad de Agronomía, Universidad de Buenos Aires, Ciudad de Buenos Aires, Av. San Martín 4453, Argentina; ccrocco@agro.uba.ar (C.D.C)
[3] Instituto de Ciencias Biológicas, Universidad de Talca, Av. Lircay S/N, Talca 3460000, Chile; marco.molina@utalca.cl (M.A.M-M)
[4] Centro de Estudios Avanzados en Zonas Áridas, Universidad Católica del Norte, Coquimbo, Ossandón 877, Chile
[5] Centro de Investigación en Estudios Avanzados del Maule, Universidad Católica del Maule, Talca, Av. San Miguel 3605, Chile
* Correspondence: lbubica@agro.uba.ar; Tel.: +54-11-4-524-8000

Supplementary Materials: The following are available online at www.mdpi.com/xxx/s1.

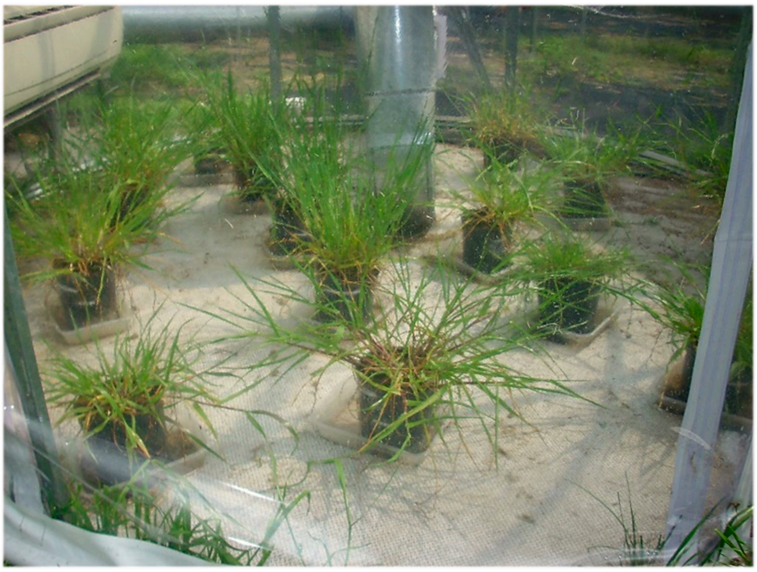

Figure S1. Inside view of an open-top tropospheric ozone chamber with the *Lolium multiflorum* mother plants during the ozone exposure treatment.

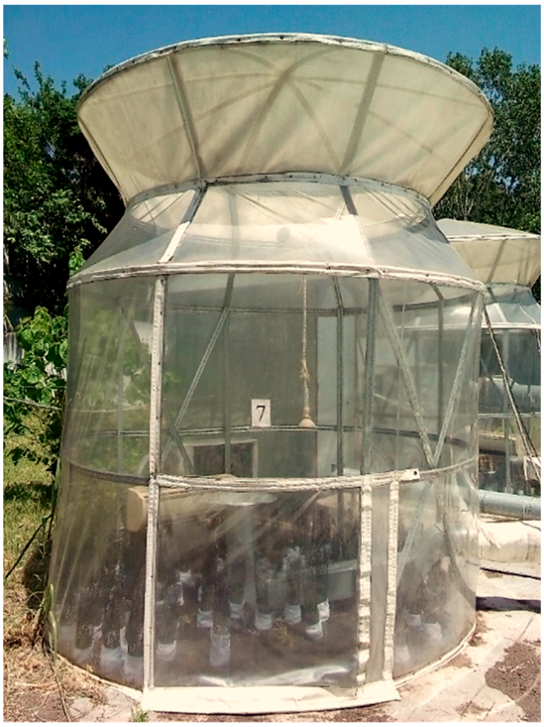

Figure S2. View of two of the eight open-top chambers used for the controlled ozone exposure of *Lolium multiflorum* plants in the experiment.

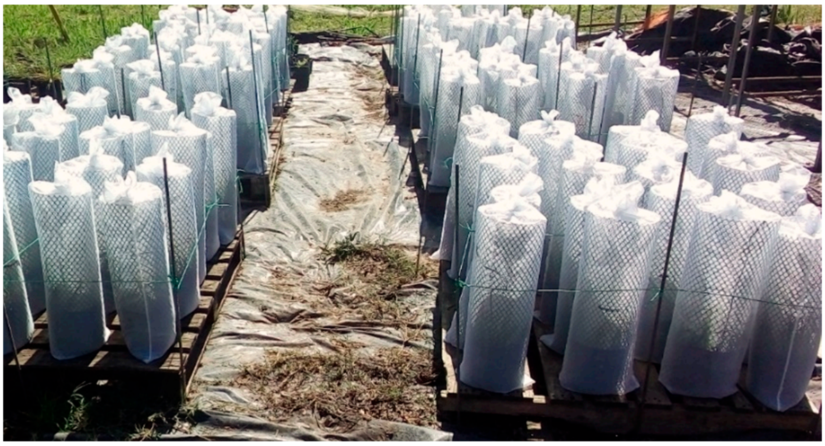

Figure S3. View of the experimental setting where *Lolium multiflorum* plants with different symbiotic status and maternal ozone history were challenged with the aphid *Rhopalosiphum padi* during the herbivory treatment. Plants were surrounded with a plastic net cylinder and covered by a white fabric.

Table S1. Significance of effects of the endophyte symbiosis and mother plant exposure to ozone on response variable "number of aphids" over *Lolium multiflorum* progeny plants. Statistically significant effects are highlighted in bold. Mean values and S.E. and statistical differences are shown in Figure 2.

Response variable	Treatments	χ^2	P-value
Number of aphids (#. Pl $^{-1}$)	Maternal ozone	1.8448	0.174389
	Endophyte	10.3723	**0.001279**
	Maternal ozone x Endophyte	3.0597	0.080256

Table S2. Significance of effects of the endophyte symbiosis and mother plant exposure to ozone on response variable "proportion of individuals per instar" over *Lolium multiflorum* progeny plants. Statistically significant effects are highlighted in bold. Mean values and S.E. and statistical differences are shown in Figure 2.

Response variable	Treatments	χ^2	P-value
Proportion of individuals per instar (#. Pl $^{-1}$)	Maternal ozone	0.001	0.9572
	Endophyte	9.600	**0.0019**
	Maternal ozone x Endophyte	0.220	0.6394

Table S3. Significance of effects of the endophyte symbiosis and mother plant exposure to ozone on response variable "aphid individual weight" over *Lolium multiflorum* progeny plants. Statistically significant effects are highlighted in bold. Mean values and S.E. and statistical differences are shown in Table 1.

Response variables	Treatments	df	F-value	P-value
Aphid individual weight: nymphs (μg. individual $^{-1}$)	Maternal ozone	1.6	2.4395	0.1693
	Endophyte	1.67	8.7628	**0.0042**
	Maternal ozone x Endophyte	1.67	6.6301	**0.0122**
Aphid individual weight: apterous (μg. individual $^{-1}$)	Maternal ozone	1.6	0.6108	0.4642
	Endophyte	1.65	6.1724	**0.0156**
	Maternal ozone x Endophyte	1.65	4.0712	**0.0478**
Aphid individual weight: winged (μg. individual $^{-1}$)	Maternal ozone	1.6	0.1343	0.7266
	Endophyte	1.37	0.0103	0.9199
	Maternal ozone x Endophyte	1.37	1.7020	0.2001

Table S4. Significance of effects of the endophyte symbiosis and mother plant exposure to ozone on response variable "total phenolic compounds" over *Lolium multiflorum* progeny plants. Statistically significant effects are highlighted in bold. Mean values and S.E. and statistical differences are shown in Figure 3.

Response variables	Treatments	df	F-value	P-value
Total phenolic compounds from seeds (at 320 nm)	Maternal ozone	1.6	8.2108	**0.0286**
	Endophyte	1.38	5.2591	**0.0275**
	Maternal ozone x Endophyte	1.38	0.0398	0.8430
Total phenolic compounds from progeny plants (at 320 nm)	Maternal ozone	1.6	0.2113615	0.6619
	Endophyte	1.74	1.5125059	0.2227
	Maternal ozone x Endophyte	1.74	0.7755067	0.3814

Table S5. Significance of effects of the endophyte symbiosis and mother plant exposure to ozone on response variable "shoot biomass" over *Lolium multiflorum* progeny plants. Statistically significant effects are highlighted in bold. Mean values and S.E. and statistical differences are shown in Figure 4.

Response variables	Treatments	df	F-value	P-value
Shoot biomass (g. DW. Plant $^{-1}$)	Maternal ozone	1.6	0.05427	0.8235
	Endophyte	1.68	11.76128	**0.001**
	Maternal ozone x Endophyte	1.68	0.6306	0.4299
